# Influence of* Helicobacter pylori* Infection on Metabolic Syndrome in Old Chinese People

**DOI:** 10.1155/2016/6951264

**Published:** 2016-06-27

**Authors:** Wen Yang, Cunfu Xuan

**Affiliations:** ^1^Geriatric Digestive System Department, Navy General Hospital, No. 6 Fuchenglu Road, Beijing 100048, China; ^2^Department of Cardiology, Shuozhou People's Hospital, No. 263 ShanYang Street, Shuocheng District, Shuozhou, Shanxi 036002, China

## Abstract

*Background. H. pylori* infection is one of the most common chronic infectious inflammatory diseases worldwide and is also a risk factor for atherosclerosis. Patients with metabolic syndrome are known to be at increased risk for atherosclerosis. The aim of our study was to assess the effects of* H. pylori* infection on serum lipids, body mass index (BMI), and metabolic syndrome in old Chinese people.* Material and Method.* A total of 191 (133 males and 58 females, aged 73.19 ± 11.03 years) people who had gastroscopy examination in our hospital were divided into* H. pylori*-positive group (*n* = 80) and* H. pylori*-negative group (*n* = 111).* H. pylori* infection was diagnosed by rapid urease test.* Results.* Patients with* H. pylori* infection had higher BMI and fasting glucose levels and incidence of metabolic syndrome (*p* < 0.01). It was found that BMI (*p* < 0.01, OR 74.469),* H. pylori* infection (*p* < 0.01, OR 5.427), total cholesterol (*p* < 0.01, OR 15.544), and diabetes mellitus (*p* < 0.01, OR 23.957) were significantly associated with the risk of metabolic syndrome by binary logistic regression analysis.* Conclusions.* Patients with* H. pylori* infection had higher BMI and fasting glucose levels and had incidence of metabolic syndrome.

## 1. Introduction

*H. pylori*, a Gram-negative bacterium that dwells on the gastric epithelium, infects over 50% of the human population, with a high rate in those living in developing countries [1].* H. pylori* can cause many gastrointestinal diseases, including peptic ulcers, chronic gastritis, and gastric mucosa-associated lymphoid tissue lymphoma (MALToma). It is also considered a class I carcinogen that can induce chronic inflammation and gastric cancer [2, 3].

In recent years, several studies demonstrated that the outcome of* H. pylori* infection may not be confined to the digestive tract, and that the infection can be associated with extradigestive pathologies including atherosclerotic vascular diseases [4–6]. Atherosclerosis is a multifactorial disease.* H. pylori* may disturb lipid and glucose metabolism in a way that may increase the risk of atherosclerosis [7].

Metabolic syndrome has become a worldwide public health issue, and it is also a risk factor for atherosclerosis. According to the National Cholesterol Education Program Adult Treatment Panel III (NCEP ATP III), metabolic syndrome is composed of the following major components: abdominal obesity, insulin resistance (IR), elevated BP, and dyslipidemia [8].

This study aimed to determine the prevalence of metabolic syndrome and its components in* H. pylori*-positive patients in middle-aged and elderly Chinese population.

## 2. Material and Method

### 2.1. Study Population and Data Collection

This study was conducted at Navy General Hospital. All qualified subjects who attended their annual health examination during the year of 2014 were initially enrolled. Inclusion criteria were complete, available medical records and having gastroscopy in our hospital; having not used proton pump inhibitors (PPIs), histamine type 2 receptor antagonists (H_2_A), antibiotics, bismuth, or sucralfate for up to one month prior to the endoscopy; stopping using various anticoagulation and antiplatelet drugs for more than a week; normal blood pressure (diastolic blood pressure (DBP) of less than 90 mmHg and systolic blood pressure (SBP) of less than 150 mmHg) or well-controlled hypertension (DBP of less than 90 mmHg and SBP of less than 150 mmHg). Exclusion criteria were the presence of hematological diseases; past history of cancer; major gastrointestinal surgery, including partial or total gastrectomy or colectomy; pulmonary disease; smoking; alcohol consumption; nephrotic syndrome or serum creatinine levels higher than 422 *μ*mol/L; having been using the medicine which is known to be able to effect blood coagulation, such as aspirin and clopidogrel; platelet count below 100000/mL; previously being diagnosed with anemia. The definition of anemia in this study was serum hemoglobin < 120 g/L for males and <110 g/L for females. Finally, 191 eligible subjects were enrolled (133 males and 58 females, aged 73.19 ± 11.03 years). Written informed consent was obtained from each subject and was recorded by the physician who explained the study procedures. The study was reviewed and approved by the Ethics and Research Committee of the Navy General Hospital (Beijing, China) and the reported investigations were carried out in accordance with the principles of the Declaration of Helsinki as revised in 2000.

### 2.2. Metabolic Syndrome Diagnosis

Metabolic syndrome was diagnosed following the China Adult Dyslipidemia Prevention Guide on metabolic syndrome [9]. The subject was in accordance with three or four of the following criteria: (1) overweight and/or obesity: body mass index (BMI) > 25.0 kg/m^2^; (2) hyperglycemia: fasting blood glucose (FBG) ≥ 6.1 mmol/L or 2 h postmeal plasma glucose >7.8 mmol/L or having been diagnosed with diabetes mellitus; (3) hypertension: SBP ≥ 140 or DBP ≥ 90 mmHg or taking ≥1 antihypertensive agent; (4) dyslipidemia: fasting triglycerides (TGs) ≥ 1.7 mmol/L and/or high-density lipoprotein cholesterol (HDL-C) ≤ 0.9 mmol/L for males and ≤1.0 mmol/L for females.

### 2.3. Clinical Examination

All of the subjects were interviewed regarding current health status (diabetes mellitus, hypertension, and gastrointestinal diseases) and were asked not to do exercise for one day prior to the medical examination. Blood pressure was measured in the right arm using a mercury sphygmomanometer after 20 min of rest with the participants in a sitting position. The first and fifth Korotkoff sounds were used as systolic and diastolic blood pressure. Standing height, body weight, and waist circumference were recorded for all subjects. Waist circumference was measured with the measuring tape positioned midway between the lowest rib and the superior border of the iliac crest as the participants exhaled normally. BMI was calculated as weight divided by height squared.

### 2.4. Biochemical Analyses

Blood samples were taken into anticoagulated tubes from participants after an overnight fast of more than 12 h. Plasma separated by centrifugation at 3000 ×g for 10 minute at room temperature. The levels of creatinine, BUN, total cholesterol, triglyceride, and glucose were measured using a multichannel analyzer (Roche Hitachi 737; Boehringer Mannheim Diagnostics, USA).

### 2.5. Gastroscopy and* H. pylori* Examination

All subjects were required to refrain from intake of food and water on the morning of gastroscopy, and gastroscopy was performed routinely under light intravenous sedation and local anesthetic spray to the oropharynx. A diagnosis of* H. pylori* infection was made if* H. pylori *morphology was seen on histopathological examination and the rapid urease test during gastroscopy was positive. Patients with negative results in one or both examinations were considered to be* H. pylori*-negative.

The gastroscopic procedures were performed using an upper gastrointestinal video endoscope (Olympus EVIS EXERA III, CV-190). The whole stomach was examined first with conventional endoscopy. After the whole stomach mucosa was observed the sites were chosen for biopsy of the gastric mucosa. The biopsy forceps were taken from the distal helicobacter 1-2 cm mucous membrane and then put it in the urease test wells for* H. pylori* quick test (Biohit Plc., Helsinki, Finland). The exact time of the placement of the biopsies in the urease test wells was recorded and the wells were inspected for color change at 2 min, 30 min, 2 h, and 24 h. The test was assigned positive when there was a color change of at least 2 mm radius of red cloud around the biopsy specimen or complete color change of the yellow well to red or magenta; negative color stayed the same. At the same time, a piece of gastric mucous membrane specimen was taken for pathologic examination. The gastric tissue specimens were submitted to the pathologist for histological analysis. The hematoxylin-eosin and the Giemsa stainings were used for identification of* H. pylori*. To minimize the potential bias, the pathological analysis was made by one experienced pathologist at Pathological Laboratory of Navy General Hospital.

### 2.6. Statistical Analysis

Data were expressed as mean ± SD or counts. Statistical analysis was performed using SPSS version 16.0 (SPSS Inc., Chicago, IL), and the level of statistical significance was defined as *p* < 0.05. The independent samples *t*-test was used for the comparisons of continuous data, while the chi-square test was used for the comparisons of categorical variables. Binary logistic regression analysis was used to determine the factors that were associated with metabolic syndrome.

## 3. Results

### 3.1. Baseline Characteristics

Among the 191 enrolled patients (133 males and 58 females, aged 73.19 ± 11.03 years), 80 (59 males and 21 females) were diagnosed with* H. pylori* infection. The prevalence of* H. pylori* infection was 41.89% (males 44.36% and females 36.21%). The characteristics of the patients, classified being* H. pylori-*positive or* H. pylori-*negative, are presented in Table 1. Patients with* H. pylori* infection had higher BMI and fasting glucose levels and incidence of metabolic syndrome (*p* < 0.01).

### 3.2. *H. pylori* Infection and Risk Factors for Metabolic Syndrome

Binary logistic regression analysis was used to evaluate the risk factors for metabolic syndrome. Metabolic syndrome was taken as the dependent variable and age, gender, SBP, DBP, BMI,* H. pylori* infection, total cholesterol, triglyceride, fasting glucose, creatinine, BUN, hypertension, and diabetes mellitus were taken as independent variables. It was found that BMI (*p* < 0.01, OR 74.469),* H. pylori* infection (*p* < 0.01, OR 5.427), total cholesterol (*p* < 0.01, OR 15.544), and diabetes mellitus (*p* < 0.01, OR 23.957) were significantly associated with the risk of metabolic syndrome (Table 2).

## 4. Discussion

This study showed a positive association between* H. pylori* infection and the prevalence of metabolic syndrome among a group of subjects from middle-aged to elderly Chinese population, which is in agreement with the previous studies [10, 11]. According to the multiple logistic regression analyses performed in this study,* H. pylori* infection was found to be associated with an increased risk of metabolic syndrome, indicating that* H. pylori* infection could be used as a risk factor of metabolic syndrome.

The mechanisms underlying the association between* H. pylori* infection and metabolic syndrome and its role in predicting metabolic syndrome in obese patients are unclear. There are three possible mechanisms that might explain our findings. First,* H. pylori* infection impairs secretion balance of proinflammatory cytokines and CRP, angiotensinogen, free fatty acids, and leptin hormone, and thus, reactive oxygen species begin to accumulate. Subclinical chronic inflammation induced by* H. pylori *infection occurs via impaired cytokine balance and stimulated macrophages [12, 13]. There are explanations that this leads to unresponsiveness to insulin in the peripheral tissue and subsequently to metabolic syndrome [14–17]. Second, Ghrelin, as a multifunctional polypeptide secreted from gastric mucosa, is involved in ingestion, appetite, and nutrition, especially lipid absorption and lipogenesis [18, 19]. The Ghrelin can also modulate insulin sensitivity and stimulate insulin-induced glucose uptake [20], and the* H. pylori* infection can impair Ghrelin synthesis [21]. Third, the previous studies showed that infection with* H. pylori* had a positive association with high LDL, low HDL, and cardiovascular disease and successful* H. pylori *eradication decreased the risk of high LDL and low HDL [22].

In short, metabolic syndrome is significantly increased in patients with* H. pylori* infection, which will help to explore the pathogenesis of the metabolic syndrome. It is necessary to further research the relationship between* H. pylori* infection and metabolic syndrome. We believe that* H. pylori* eradication has the potential to be used in prevention and treatment of the metabolic syndrome.

A few limitations warrant consideration. First, we did not investigate the patients with* H. pylori* infection after treatment. Second, this was a single-center study and thus our relatively small sample size may have posed a limitation to this study. Therefore, our findings need to be confirmed in multicenter and prospectively designed studies. Third, we did not examine* H. pylori *infection by urea breath test or stool antigen test, which cannot exclude the past infection; that is, in case the patients had severe atrophy,* H. pylori* cannot be detected there. Finally, we did not investigate the Ghrelin or proinflammatory cytokines, which could confound the pathogenesis of the metabolic syndrome.

## Figures and Tables

**Table 1:** Characteristics of study subjects according to the *H. pylori* infection.

Variables	*H. pylori *negative (*n* = 111)	*H. pylori *positive (*n* = 80)	*p* value
Age	71.89 ± 11.07	75.00 ± 10.80	0.055
Male, *n* (%)	74 (66.67)	59 (73.75)	0.295
SBP (mmHg)	132.79 ± 13.33	131.58 ± 14.19	0.547
DBP (mmHg)	74.06 ± 8.23	75.74 ± 9.66	0.200
BMI (kg/m^2^)	23.10 ± 2.74	24.31 ± 2.70	0.003
Metabolic syndrome, *n* (%)	42 (37.84)	43 (53.75)	0.001
Total cholesterol (mmol/L)	4.22 ± 1.15	4.36 ± 0.88	0.383
Triglycerides (mmol/L)	1.34 ± 0.81	1.21 ± 0.52	0.221
Fasting glucose (mmol/L)	5.66 ± 1.40	6.20 ± 1.80	0.022
Creatinine (*μ*mol/L)	101.54 ± 34.79	100.65 ± 24.23	0.845
BUN (mmol/L)	6.03 ± 2.26	6.27 ± 1.90	0.443
Hypertension, *n* (%)	32 (28.83)	19 (23.75)	0.435
Diabetes mellitus, *n* (%)	19 (17.12)	21 (26.25)	0.135

**Table 2:** The results of binary logistic regression analysis on metabolic syndrome.

	Variable	SE	Beta	*p* value	OR	95.0% CI	
						Lower	Upper
Age	<65 = 0, ≥65 = 1	0.674	0.503	0.485	1.602	0.428	5.999
Male, *n*	Female = 0, male = 1	0.582	−0.066	0.837	0.887	0.283	2.776
SBP (mmHg)	<140 = 0, ≥140 = 1	0.623	0.602	0.216	2.161	0.637	7.324
DBP (mmHg)	<90 = 0, ≥90 = 1	0.909	0.831	0.290	2.619	0.441	15.551
BMI (kg/m^2^)	<25 = 0, ≥25 = 1	0.683	4.117	0.001	74.469	19.507	284.29
*H. pylori* infection (*n*)	Absent = 0, present = 1	0.575	1.538	0.003	5.427	1.757	16.76
Total cholesterol (mmol/L)	<5.69 = 0, ≥5.69 = 1	0.945	2.620	0.004	15.544	2.441	98.981
Triglycerides (mmol/L)	<1.7 = 0, ≥1.7 = 1	0.704	0.243	0.797	0.834	0.21	3.316
Fasting glucose (mmol/L)	<6.1 = 0, ≥6.1 = 1	0.959	1.392	0.091	5.053	0.772	33.068
Creatinine (*μ*mol/L)	<115 = 0, ≥115 = 1	0.796	1.076	0.258	2.46	0.517	11.703
BUN (mmol/L)	<8.2 = 0, ≥8.2 = 1	0.91	0.237	0.873	0.865	0.145	5.151
Hypertension (*n*)	Absent = 0, present = 1	0.679	−0.111	0.910	0.926	0.245	3.504
Diabetes mellitus (*n*)	Absent = 0, present = 1	1.202	3.182	0.008	23.957	2.271	252.722
